# The Next Influenza Wave

**Published:** 1919-10-11

**Authors:** 


### THE NEXT INFLUENZA WAVE.

Whatever may be the particular circumstances with which we can correctly associate the onset of influenza outbreaks, there is no doubt that within the past fortnight there has been a definite reappearance of widespread catarrhs such as were too often seen last year. They have been relatively mild cases so far, and not usually followed by any serious complications, but it is certain that we still have this infection smouldering among the population, particularly in the greater cities. The number of acutely septic throats which have occurred in certain localised areas, showing cultural and clinical characters identical with those of 1918, is more than suggestive that our fears of a recrudescence this winter may be fully realised.

Several journals have called up the examples in the recently published report on the influenzal mortality in Scotland, where 17,573 deaths occurred during the last waves, and they remind us gently of danger ahead. It is hardly necessary, however, to adopt any excuse for drawing public attention to an obvious menace. As yet no new outbreak has seriously begun, the great majority of people still cannot have encountered the infection, and therefore the present is the exact time at which both the public and the medical profession can most advantageously institute early prophylactic measures.

The question of vaccination is still dividing the profession. A very large number of authorities advovate protective inoculation, although even they will admit there is an element of empiricism in the procedure. Our own opinion and experience is that considerable protection against the complications of the disease may be obtained by vaccine administration, and inasmuch as it is these complications, commonly pneumo- or strepto-coccal in origin, which are the cause of the greater part of the seriousness to be attached to an epidemic, we would strongly advise the use of this protective method in all adults, particularly those between ages of twenty and forty years. It was within these limits that the greatest mortality occurred, in spite of the relatively robust health enjoyed in the majority of instances, but in stating such advice we would call attention to the fact that inoculation is followed by a negative phase of about two days, in which the patient is more susceptible to infection, and it is thus risky to administer a dose either during an indisposition or at a time when it is impossible for the next few hours to take relatively good care of the patient from the infection point of view at least.

It is out of the question in these few lines to touch even briefly upon the *pros* and *cons* of times, dosage, and constituents. All we can do is to suggest, on considerable first-hand experience, that *before* influenza becomes again rampant throughout the land the public should submit to protective inoculation, and that the profession should take pains to obtain, fully to understand, and to use the prophylactic vaccines which are now very readily obtainable from the finest laboratories in the country.

There are many who will tell us that to talk of prepared antitoxin and artificial immunity in a disease of unknown etiology is more than a little illogical. True, it is that the wide use of these inoculations has been to some extent experimental, but we at least know that those intended to accentuate the bodily resistance against a possible pneumonia have frequently been of value if the pneumonia did develop, and harmless if it did not.

There is also one other point to urge. In the past the public press has been full to overflowing with general health directions, protective and preventive, for the people's benefit, but we should realise by experience that once the epidemic has started most of these measures are of regrettably little avail. There is no need for scares and anxiety, particularly in such troubled times as the present, but, nevertheless, the public might well be urged *now* to adopt the simple principles of hygiene and care of the throat and respiratory passages which were so loudly but belatedly called for last winter.

